# Overcoming immuno-resistance by rescheduling anti-VEGF/cytotoxics/anti-PD-1 combination in lung cancer model

**DOI:** 10.20517/cdr.2023.146

**Published:** 2024-03-14

**Authors:** Guillaume Sicard, Dorian Protzenko, Sarah Giacometti, Fabrice Barlési, Joseph Ciccolini, Raphaelle Fanciullino

**Affiliations:** ^1^SMARTc Unit, CRCM Inserm U1068, Aix Marseille University, Marseille 13385, France.; ^2^School of Medicine, Aix Marseille University, Marseille 13385, France.; ^3^Department of Thoracic Oncology, Gustave Roussy Institute, Villejuif 94200, France.; ^4^COMPO, CRCM Inserm U1068 INRIA, Marseille 13385, France.

**Keywords:** Immune checkpoint inhibitors, cisplatin, pemetrexed, anti-VEGF, non-small cell lung cancer, efficacy study, immunomonitoring

## Abstract

**Background:** Many tumors are refractory to immune checkpoint inhibitors, but their combination with cytotoxics is expected to improve sensitivity. Understanding how and when cytotoxics best re-stimulate tumor immunity could help overcome resistance to immune checkpoint inhibitors.

**Methods:** *In vivo* studies were performed in C57BL/6 mice grafted with immune-refractory LL/2 lung cancer model. A longitudinal immunomonitoring study on tumor, spleen, and blood after multiple treatments including Cisplatin, Pemetrexed, and anti-VEGF, either alone or in combination, was performed, spanning a period of up to 21 days, to determine the optimal time window during which immune checkpoint inhibitors should be added. Finally, an efficacy study was conducted comparing the antiproliferative performance of various schedules of anti-VEGF, Pemetrexed-Cisplatin doublet, plus anti-PD-1 (i.e., immunomonitoring-guided scheduling, concurrent dosing or a random sequence), as well as single agent anti-PD1.

**Results:** Immunomonitoring showed marked differences between treatments, organs, and time points. However, harnessing tumor immunity (i.e., promoting CD8 T cells or increasing the T CD8/Treg ratio) started on D7 and peaked on D14 with the anti-VEGF followed by cytotoxics combination. Therefore, a 14-day delay between anti-VEGF/cytotoxic and anti-PD1 administration was considered the best sequence to test. Efficacy studies then confirmed that this sequence achieved higher antiproliferative efficacy compared to other treatment modalities (i.e., -71% in tumor volume compared to control).

**Conclusions:** Anti-VEGF and cytotoxic agents show time-dependent immunomodulatory effects, suggesting that sequencing is a critical feature when combining these agents with immune checkpoint inhibitors. An efficacy study confirmed that sequencing treatments further enhance antiproliferative effects in lung cancer models compared to concurrent dosing and partly reverse the resistance to cytotoxics and anti-PD1.

## INTRODUCTION

Lung cancer is the second most frequent cancer^[1]^ and the leading cause of cancer death^[2]^. Non-small cell lung cancer (NSCLC) accounts for around 80% to 85% of all lung cancers, which are divided into three main types: adenocarcinoma, squamous cell carcinoma, and large cell carcinoma^[3]^. According to NICE guidelines, in the absence of actionable targets such as EGFR mutations, chemotherapy of stage IIIB/IV (i.e., advanced or metastatic disease) should be based upon a doublet including a platinum derivative plus an immune checkpoint inhibitor (ICI)^[4]^. The alkylating agent Cisplatin is usually administered in combination with the antifolate Pemetrexed, augmented with immune checkpoint inhibitors such as pembrolizumab, nivolumab (i.e., anti-PD-1) or atezolizumab or durvalumab (i.e., anti-PD-L1)^[5]^.

ICIs have been considered breakthrough therapies. However, after an initial phase of groundbreaking progress in diseases with poor prognoses such as metastatic melanoma or advanced NSCLC, the 5-year survival rates are now 30%-40% at best, necessitating the development of new approaches such as combination with other treatments to further improve their use and overcome resistance^[6]^. Although first-in-class ipilimumab was originally approved as a single agent^[7]^, it is considered now that ICIs are to be combined with other therapeutic strategies, such as chemotherapy, targeted therapy, anti-angiogenics, or radiotherapy, to increase their efficacy.

Due to the almost infinite number of possible combinations to be tested in terms of scheduling and sequencing, which would require an unsustainable effort in terms of non-clinical and clinical development, many combination studies have been formulated empirically, such as incorporating ICI into a previously cytotoxic regimen. However, non-clinical studies have shown that optimizing the scheduling of ICIs can lead to spectacular results, thus advocating the search for new protocols that optimize dosing, scheduling, and sequencing^[8,9]^. Successful combinatorial strategies with immune checkpoint inhibitors rely on using cytotoxics that are most likely to harness tumor immunity, but once identified, determining the best way to further combine them with an immune checkpoint inhibitor is yet to be established to reduce resistance^[10]^.

To achieve this, it is essential to get a thorough picture of the impact of a drug on tumor immunity over time, in particular, to better understand whether there is a window of opportunity when tumors that were previously considered “cold” from an immunological point of view transition to a “hot” state - coinciding with the best time to add an immune checkpoint inhibitor whose efficacy depends on the presence of active immune cells in the tumor microenvironment.

The immunomodulatory properties of anticancer agents appear to be dependent on dose and schedule^[11]^. Cisplatin has been described to increase activated T cells and their intratumoral infiltration, whereas Pemetrexed has been found to increase intratumoral T cells^[12,13]^. One of the main issues explaining the lack of efficacy of ICIs is the fact that some tumors are immunologically “cold”, i.e., with few activated CD8 T cells and many Tregs, which downregulate the tumor immune response. The use of cytotoxic drugs could help to turn cold tumors into hot ones, firstly by inducing immunogenic cell death^[14]^, and secondly by remodeling the tumor immune landscape, i.e., by increasing the number of CD8 T cells while decreasing the number of Treg lymphocytes^[15]^. Here, we investigated the extent to which the Pemetrexed/Cisplatin doublet, with or without anti-VEGF, could harness tumor immunity over time to determine the best time window for the addition of anti-PD-1 therapy. The cell populations studied were B cells, global T cells (defined as CD3+), CD4 and CD8 T cells, Tregs, and both granulocytic-myeloid-derived suppressor cells (Gr-MDSCs) and monocytic-myeloid-derived suppressor cells (Mo-MDSCs).

These populations were studied in three different organs: blood, spleen, and tumor.

Spleen is the largest secondary lymphoid organ in the human body, accounting for about 15.2% of the total number of T cells. It allows T cell activation for antigens present in the bloodstream. Indeed, unlike other lymphoid organs, spleen is not a lymphatic organ, that is, it is not in direct contact with the lymphatic circulation but with the blood circulation.

As mentioned above, tumor-infiltrating lymphocytes (TILs) play an important role in immunotherapy response and then in immune resistance and have been defined as a factor of good prognosis.

## METHODS

To this end, our study was based on two steps: - Observe the immunomodulatory impacts of Cisplatin and Pemetrexed when administered individually at different doses (i.e., low dose and MTD) and in combination with anti-VEGF to determine the optimal therapeutic window for the addition of ICI; - To compare the efficacy of different schedules of Cisplatin, Pemetrexed, and anti-VEGF in combination with anti-PD-1 with different schedules to test whether the best sequence suggested previously by immunomonitoring achieves higher efficacy.

### Animal ethics statement

This study adhered to the French protocols for animal welfare and the 2010/63/EU mandate of the European Parliament, receiving endorsement from both the ethics committee at Aix-Marseille University and the Ministère de l’Enseignement Supérieur, de la Recherche et de l’Innovation. The procedures were documented under registration number 2021082510559854, and mice were vigilantly observed for behavioral and physical alterations by qualified personnel authorized for animal experimentation by the French Direction Départementale des Services Vétérinaires.

### Mice and cell lines

Six-week-old female C57BL/6 mice were purchased from Charles River, France. Experiments were performed on canonical and immune-refractory mice lung cancer cell line LL/2 (American Type Culture Collection, Rockville, MD, USA). Lewis lung carcinoma was a cell line established from the lung of a C57BL/6 mouse bearing a tumor resulting from an implantation of primary Lewis lung carcinoma. Cells were maintained in DMEM culture media with 10% FBS, 1% penicillin, and 0.16% kanamycin in a humidified 5% CO_2_ incubator at 37 °C.

Cells were routinely verified based on cell viability, morphology, and doubling time.

Mice were ectopically grafted on the right flank with 1 × 10^6^ LL/2 cells for the 1st experiment and with 200,000 LL/2 cells for the 2nd one.

### *In vivo* studies

For the 1st immunomodulation study, 75 six-week-old female C57BL/6 mice were split into eight subsets: saline (Control, *n* = 12), full-dose Cisplatin (FD Cisplatin, i.e., 3 µg/g, *n* = 9), low-dose Cisplatin (LD Cisplatin, i.e., 0.75 µg/g, *n* = 10), full-dose Pemetrexed (FD Pemetrexed, i.e., 100 µg/g, *n* = 10), low-dose Pemetrexed (LD Pemetrexed, i.e., 25 µg/g, *n* = 8), flat-dose anti-VEGF (i.e., 10 µg/mice, *n* = 10), anti-VEGF + both full-dose cytotoxics (CT) (anti-VEGF + FD CT, *n* = 8), and anti-VEGF + low dose CT (anti-VEGF + LD CT, *n* = 8). All therapies were delivered intraperitoneally on Day 0, followed by weekly administrations for three consecutive weeks. In the combined regimens, murine anti-VEGF was administered on Day 0, with CT following on Day 2 for each cycle, based on prior research indicating an optimal two-day gap between anti-VEGF and cytotoxic treatments^[9]^.

Blood, spleen, and tumor samples were collected on Days 2, 7, 14, and 21 on 2 or 3 mice for each group. Each tumor was weighed, and tumor and spleen cells were counted.

For the 2nd efficacy study, 42 six-week-old female C57BL/6 mice were divided into six groups: saline (*n* = 7), anti-VEGF + FD CT (Day 0 anti-VEGF and Day 2 CT, *n* = 7), anti-PD-1 single agent (*n* = 7), “concurrent protocol” (Day 0 anti-VEGF and Day 2 FD CT + anti-PD-1, *n* = 7), “test protocol” (Day 0 anti-VEGF, Day 2 FD CT and Day 14 anti-PD-1, *n* = 7), “reverse protocol” (Day 0 anti-PD-1, Day 14 anti-VEGF and Day 16 FD CT, *n* = 7). Dosing was for murine anti-VEGF: 10 µg/mice, Cisplatin: 3 µg/g, Pemetrexed: 100 µg/g, and murine anti-PD-1: 50 µg/g.

**Figure 1:**
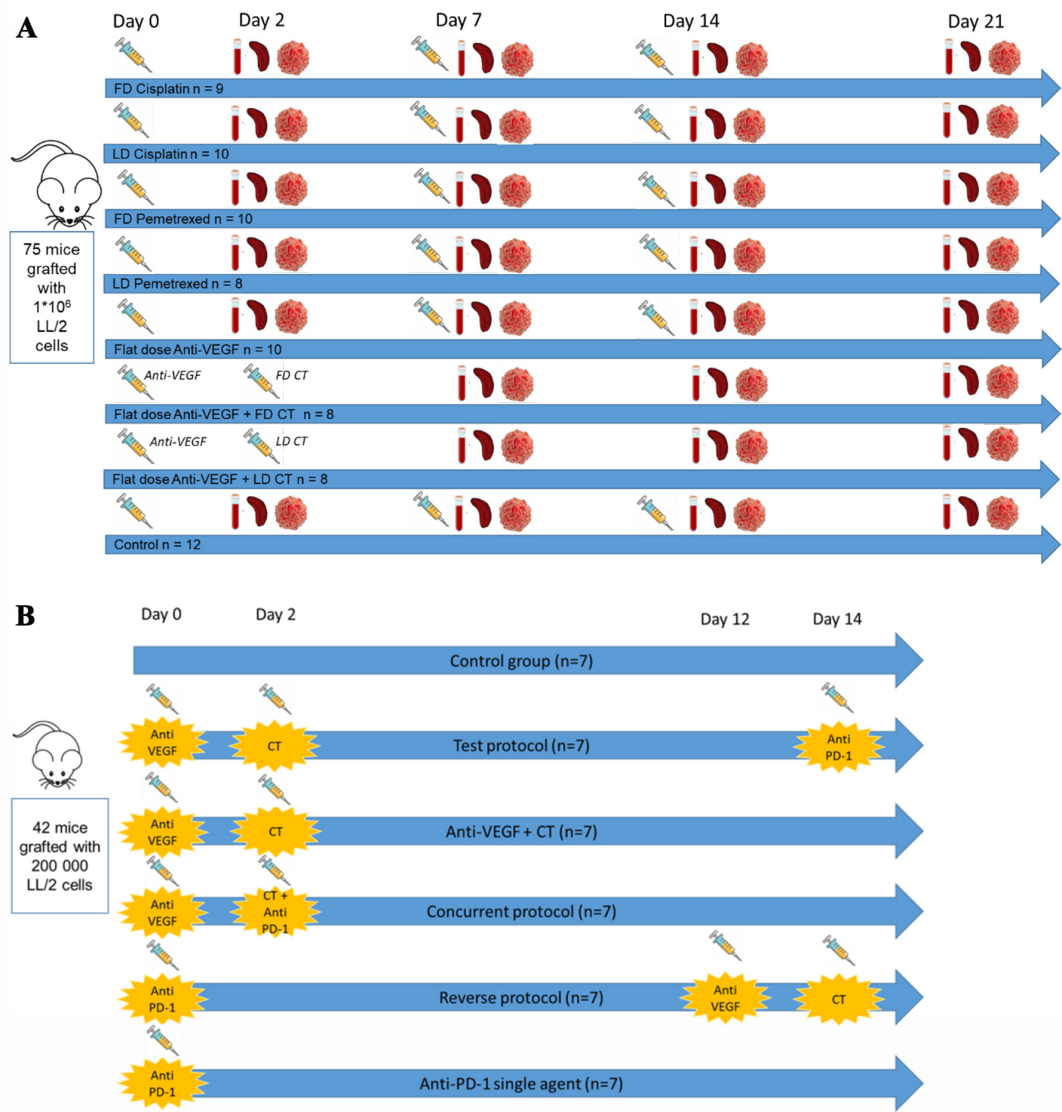
Summary of the experimental designs, (A) 1st immunomodulatory experiment; and (B) 2nd comparative efficacy experiment including anti-PD-1 treatment.

Experimental designs are summarized in Figure 1A and B.

Volumetric measurements were performed twice a week, from Day 2 to 26, using a vernier caliper. In both studies, a tumor volume larger than 1,500 mm^3^ was inacceptable and led to the sacrifice of the animal, as well as signs of pain, distress, abnormal behavior, change in food and water consumption, or loss in weight (≥ 15% from the initial weight). Daily visual examination was performed to identify such mice, and if necessary, additional assessments with a caliper were conducted to ensure that the tumor dimensions did not surpass 1,500 mm^3^, employing the conventional formula mass (g) = l wt*Pi/6, where l, w, and t denote length, width, and thickness in millimeters.

### Cell suspension preparation

Tumors and spleen were harvested every week (2 weeks after engraftment). Tumors were extemporaneously dissected into fragments followed by incubation with collagenase Type I-A from clostridium histolyticum (1 mg/mL in RPMI-2% FCS, Sigma Aldrich) for 30 min at 37 °C under agitation. Cell suspensions were resuspended by shaking every 10 min during agitation. The suspension was filtered through a 70 μm strainer. Spleen was crushed directly in RPMI, and the resultant suspension was strained through a 70 μm filter.

Red blood cells were lysed using ACK Lysis buffer before flow cytometry staining and cell isolation.

### Antibodies and flow cytometry

The following antibodies were used for flow cytometry analysis: CD45-AF700, CD25-PE, CD127-APC-eFluor780, CD3-SuperBright436, CD19-eFluor506, CD11b-PE/Cy5, Ly6C-AF488, Ly6G/Ly6C(Gr-1)-APC, Ly-6G-PE, Anti-Mouse/Rat Foxp3 FITC, and isotype control (Rat IgG2a K Isotype Control FITC) were procured from eBioscience (Waltham, MA USA); CD4-PE-Cy7 and CD8a-APC700 from BD Biosciences (San Jose, CA, USA).

Isolated cells from the spleens or tumors were incubated with anti-CD16/CD32 antibodies (BD Biosciences San Jose, CA, USA) to prevent non-specific antibody binding. Surface antigens were stained with the antibodies diluted in PBS containing 5% FCS and 2 mM EDTA and maintained at 4 °C. Intracellular stainings were performed using the Foxp3/Transcription Factor Staining Buffer Set (eBioscience, Waltham, MA, USA).

Initially, dead cells were excluded using LIVE/DEAD Fixable Aqua Dead Cell Stain (Invitrogen). All samples were fresh samples, and all cells quantified were live cells (data not shown). We decided, subsequently, not to use LIVE/DEAD Fixable Aqua Dead Cell Stain to free one more channel for use.

Multiparametric assessment was conducted utilizing a Gallios flow cytometer (Beckman Coulter, Villepinte France), with subsequent analysis executed through the Kaluza® software. Flow cytometric evaluation of immune cells was restricted to live CD45+ cells following the exclusion of doublets.

### Statistical analysis, data analysis and data visualization

Data visualization and statistical analysis were conducted utilizing Rstudio© and the ggstatsplot package^[16]^. Due to the non-parametric distribution of the data, we applied a Kruskal-Wallis one-way ANOVA for each day, each organ, and each lymphocyte population/ratio. Between-subject hypothesis testing was executed using the Dunn test, employing the Holm method for *P*-value adjustment.

Correlation was performed using a Spearman correlation test. Thus, the median was used in statistical comparison charts, whereas the mean was used for descriptive growth curves.

To increase the robustness of the statistical analysis, data imputation was performed. We modeled the ratio of the individual tumor growth with respect to each initial volume. Next, the volume was imputed according to the predicted ratio and the initial tumor volume. This method respects the variability of the imputed volumes and provides a closer approximation of the actual tumor mass. Finally, to ensure the reliability of our method, we checked that the overall consistency and trends were not altered by the imputation, and that the correlation between predicted and actual data was strong (i.e., adjusted R^2^ between 0.7 and 0.9). Once the validity of the method and the coherence of the results were checked, statistical comparisons between groups from Day 1 to 26 were performed with a *P*-value set at 0.05.

## RESULTS

### *In vivo* immunomonitoring study

No indications of toxicity were observed in mice, and there was consistent carcass weight throughout the study (data not shown). Likewise, no mice exhibited tumors surpassing 1,500 mm^3^.

Regarding the correlation between tumor size and immune cells, a linear correlation (r = 0.75) was found between CD8 T cells in blood and the number of tumor cells (*P* < 0.001, Supplementary Figure 1). No such strong correlation was found with CD45 cells (r = 0.4, data not shown). No significant difference throughout time was observed in GrMDSC between the Control group and treated animals, regardless of the treatment and the localization [Supplementary Figure 2A-C] or in MoMDSC (data not shown).

Concerning the surveillance of CD19 populations within tumors over the course of each treatment [Supplementary Figure 3A], it was observed that on Day 2, the LD Pemetrexed group showed higher levels of B cells than the FD Cisplatin subset (*P* = 0.03). Similarly, on Day 14, the flat-dose anti-VEGF group demonstrated higher B cell counts than the FD Cisplatin subset (*P* = 0.02). On Days 7 and 21, no more differences were found.

Concerning the surveillance of CD3 T populations within tumors over the course of each treatment [Supplementary Figure 3B], no difference was seen on Day 2. Regarding global T cells (CD3+), on Day 7, the FD Cisplatin group exhibited higher T cell counts than the Anti-VEGF + FD CT, Control, and LD Pemetrexed subsets (*P* = 0.03, *P* < 0.01, and *P* < 0.01, respectively). Higher T cell levels were observed in the LD Cisplatin subset than in the Anti-VEGF + FD CT, Control, and LD Pemetrexed subsets (*P* = 0.03, *P* = 0.01, and *P* < 0.01, respectively). On Day 14, the Anti-VEGF + FD CT group showed lower levels of T cells than both single-agent Cisplatin groups (*P* < 0.01 for FD Cisplatin and *P* = 0.03 for LD Cisplatin group). On Day 21, no more differences were observed.

On Day 2, no difference was observed regarding CD4 T cells in tumor [Supplementary Figure 3C]. On Day 7, the combining group anti-VEGF + LD CT showed higher levels of CD4 T cells than both the FD Cisplatin and anti-VEGF + FD CT groups (*P* < 0.001 and *P* = 0.04, respectively). No more differences were observed later on.

Tumor CD8 T cells monitoring [Supplementary Figure 3D] revealed early differences on Day 2 between the FD Cisplatin group and both the FD Pemetrexed and LD Cisplatin groups (*P* < 0.01 and *P* = 0.05, respectively). On Day 7, the Cisplatin group showed higher CD8 T cell levels than the Control group (both *P* < 0.01). The LD Cisplatin group also exhibited higher CD8 T cell counts compared to the LD Pemetrexed group (*P* = 0.02). No more differences were observed later on.

Regarding Treg, no differences were observed between each treatment in the tumor over time (data not shown). When monitoring T CD8/Treg ratio in tumor, on Day 7, a higher ratio was observed in both the FD and LD Cisplatin groups. On Day 14, a higher numerical ratio was observed in the control and FD Pemetrexed groups compared to the other groups. The differences observed on Days 7 and 14 were not seen on Day 21 [Supplementary Figure 4].

In the spleen, CD19 monitoring on Day 2 showed that B cell levels were higher in the LD Pemetrexed and flat-dose anti-VEGF groups than in the control group (*P* = 0.04 and *P* < 0.01, respectively). On Day 7, the LD Cisplatin group had higher levels of B cells than the anti-VEGF + LD CT group (*P* < 0.01). Later, no differences were observed [Supplementary Figure 5A].

Regarding global T cells (CD3+) in the spleen, on Day 2, the control group showed a higher T cell count than the flat-dose anti-VEGF group (*P* = 0.04). On Day 7, the LD Cisplatin group showed higher CD3 T cell levels than the flat-dose anti-VEGF group (*P* < 0.01) and also higher levels in the FD Cisplatin group compared to the flat-dose anti-VEGF, control, and FD Pemetrexed groups (*P* < 0.01, *P* = 0.04 and *P* = 0.05 respectively). No further differences were observed [Supplementary Figure 5B].

On Day 7, in the spleen, the combined treatment, anti-VEGF + FD CT group showed a higher presence of CD4 T cells than both the single-agent Cisplatin groups (*P* < 0.01 for FD Cisplatin group and *P* = 0.02 for LD Cisplatin group). On Day 7, the FD Pemetrexed group always showed higher CD4 T cell levels than both the single-agent Cisplatin groups (*P* < 0.01 for FD Cisplatin group and *P* = 0.02 for LD Cisplatin group). No differences were observed on Days 14 and 21. In the spleen, differences were observed between the two combination groups and the two Cisplatin monotherapy groups on Day 7 [Supplementary Figure 5C]. Indeed, CD8 T cell level was higher in the anti-VEGF + FD CT group than in the FD Cisplatin and LD Cisplatin groups (*P* < 0.01 and *P* = 0.01, respectively); and higher in the anti-VEGF + LD CT group than in the FD Cisplatin and LD Cisplatin groups (both *P* = 0.02). On Day 14, both combination groups, flat-dose anti-VEGF and LD Pemetrexed groups had numerically higher CD8 T cells than the other treatments. Later, no differences were observed [Supplementary Figure 5C and D]. For Tregs, no differences were observed between groups over time (data not shown). No differences were observed between the groups in T CD8/Treg ratios in spleen [Supplementary Figure 6].

In blood, on Day 2, the LD Cisplatin group exhibited a lower level of B cells compared to the LD flat-dose anti-VEGF and LD Pemetrexed groups (both *P* = 0.04). On Day 7, both single-agent Cisplatin groups showed lower B cell levels; in the FD Cisplatin group, the level was lower than those in the LD Pemetrexed, flat-dose anti-VEGF, and anti-VEGF + LD CT groups (*P* < 0.01, *P* < 0.01, and *P* = 0.03, respectively), and in the LD Cisplatin, the level was lower than the flat-dose anti-VEGF and LD Pemetrexed groups (*P* = 0.02 and *P* = 0.04, respectively). No differences were observed on Days 14 and 21 [Supplementary Figure 7A].

No differences were observed between treatments in terms of global T cells (CD3), T CD4 cells (Supplementary Figure 7B and C, respectively), and both CD8 T cells and Tregs in the blood (data not shown). Regarding the monitoring of the T CD8/Treg ratio in blood, on Day 2, the T CD8/Treg ratio was numerically lower in both single-agent Pemetrexed groups and in the flat-dose anti-VEGF group. On Day 7, this ratio was numerically higher in both single-agent Cisplatin groups than in the other groups. Both the combination treatment group and the flat-dose anti-VEGF group showed numerically lower T CD8/Treg ratios on Day 14. All these differences observed from Day 2 to 14 disappeared by Day 21 [Supplementary Figure 8].

**Figure 2:**
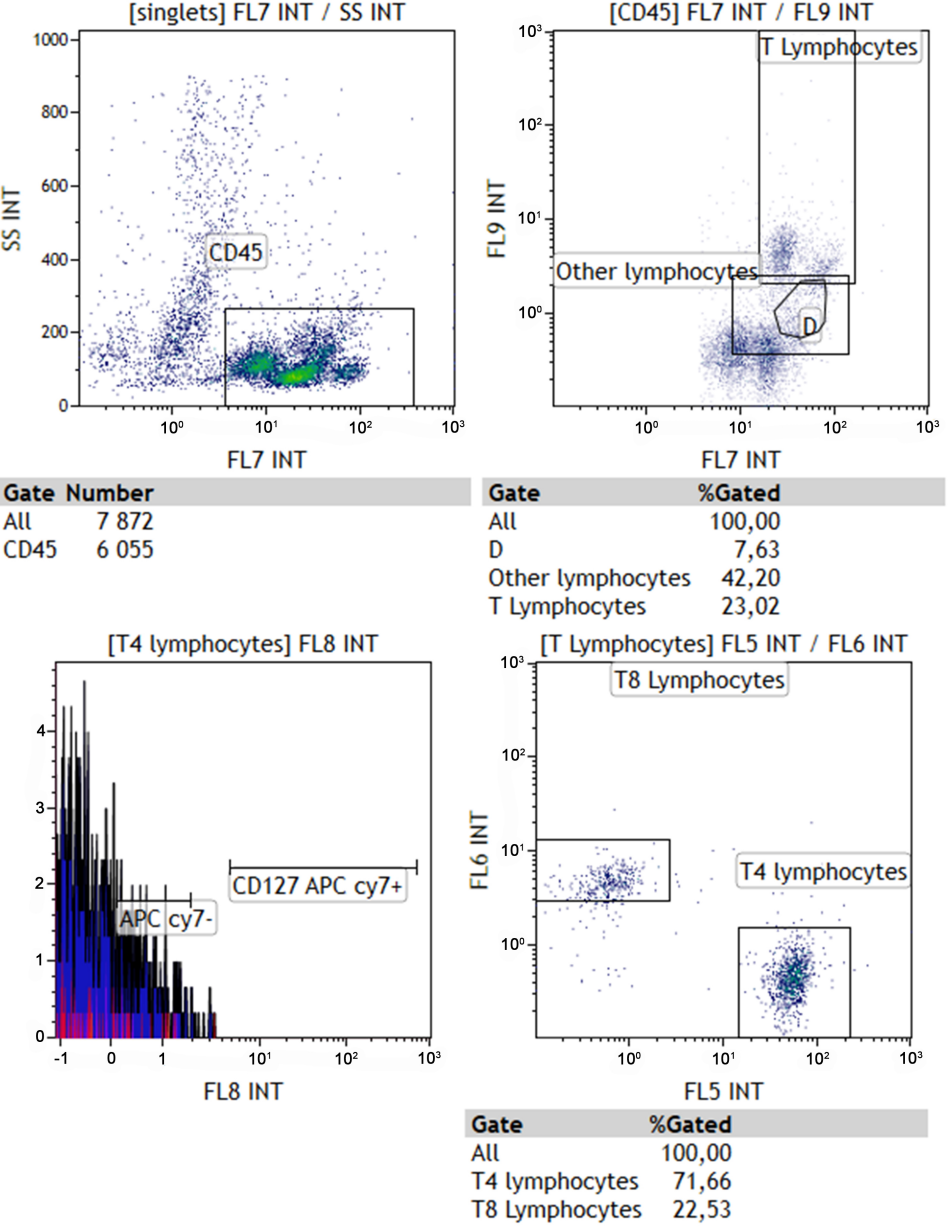
Representative screenshot of FACS analysis using the Kaluza software.

All the raw data regarding immunomonitoring are provided in Supplementary Files. Figure 2 is an example of a representative FACS analysis.

**Figure 3:**
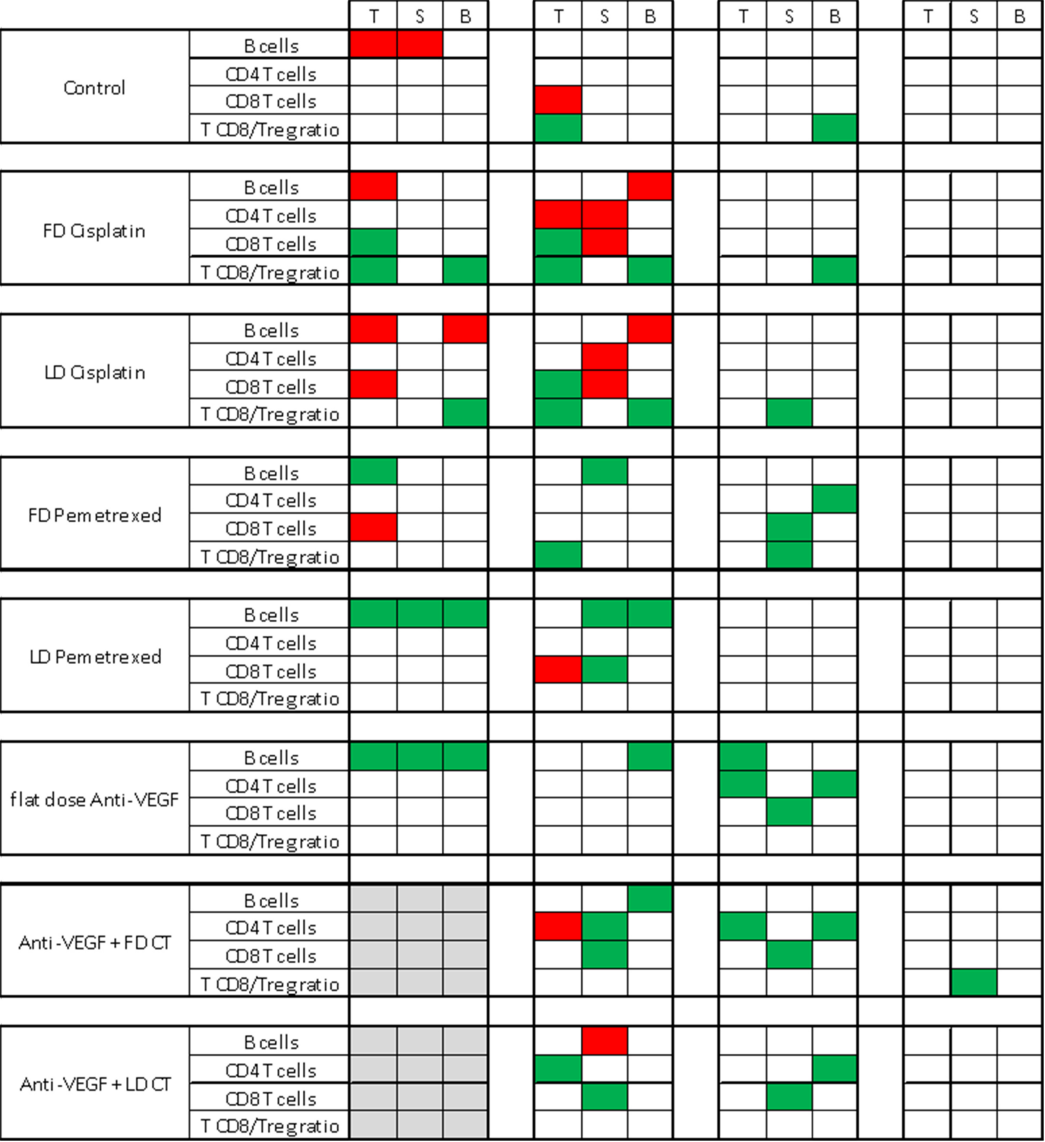
The levels of B and T cells change over time in blood, spleen, and tumor. White square: no change; Red square: decrease; Green square: increase; grey square: not tested. T: Tumor; B: Blood; S: Spleen.

Figure 3 illustrates the results regarding the monitoring of B and T cells. On Day 14, all groups, including the anti-VEGF group, showed higher T cell levels in all organs including tumors as compared with all other treatments. T CD8/Tregs ratios were higher in blood for the Control and FD Cisplatin groups. On Day 21, all the previously observed changes were normalized, with the exception of a higher T CD8/Treg ratio in spleen in the anti-VEGF + FD CT subset. Overall, the most marked changes in immune cells were noted on Day 14 in the anti-VEGF + CT FD group. The Day 0 anti-VEGF, Day 2 FD CT, and Day 14 ICI were then identified as the Test Protocol sequence to be tested next.

### *In vivo* efficacy studies

Regardless of the treatments, no toxicity was observed in mice and no difference in carcass weight was observed (data not shown).

**Figure 4:**
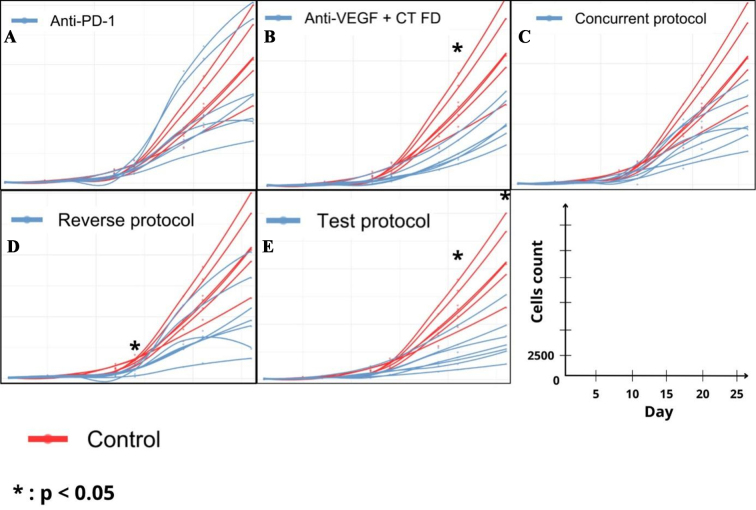
Simulation of individual growth tumor by treatment compared with control. (A) Anti-PD1; (B) Anti-VEGF + CT; (C) Concurrent Protocol; (D) Reverse Protocol; (E) Test Protocol.

**Figure 5:**
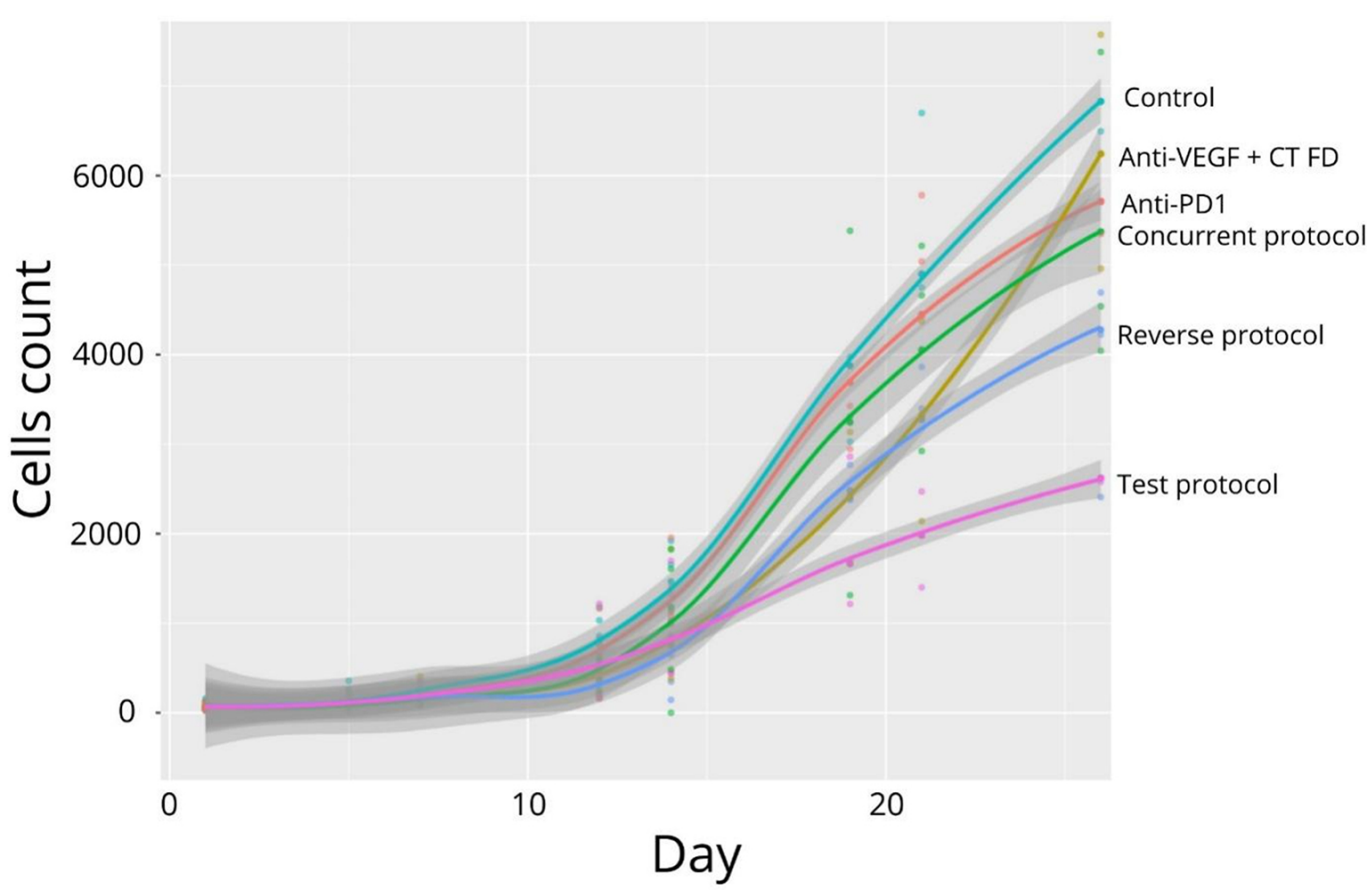
Simulation of tumor growth over 26 days (*n* = 7 per group).

Tumor growth is displayed in Figures 4 and 5. No significant differences in tumor growth were observed between the groups from Day 1 to 7. On Day 21, significantly lower tumor size was observed in the Reverse Protocol, Anti-VEGF + CT, and Test Protocol groups as compared with the Control group (*P* < 0.01, *P* = 0.03, and *P* < 0.0001, respectively). In addition, the Test Protocol group showed lower tumor size than both Concurrent protocol and Anti-PD-1 groups (*P* = 0.03 and *P* < 0.001, respectively). Finally, on Day 26, the Test Protocol group showed lower tumor size than all other groups. At the conclusion of the study, differences were significant between the Test Protocol group and Control group, Anti-VEGF + CT and Anti-PD-1 groups (*P* < 0.0004, *P* < 0.01, and *P* = 0.04, respectively), and between the Reverse Protocol group and the Control group (*P* < 0.01).

On Day 14, a significant reduction was observed between the Control group and the anti-VEGF + CT group, the Reverse protocol, and the Test protocol (-45%, - 49%, and -46%, respectively). These differences were observed up to Day 21 and we observed a significant reduction between the Control group and the anti-VEGF + CT, the Reverse protocol, the Test protocol, and also the Concurrent protocol groups (not observed on Day 14) (-61%, - 49%, -70%, and -36%, respectively). On Day 26, reduction in tumor size was more marked in the Test Protocol (-71%) than with the anti-VEGF + CT, Reverse Protocol, and Concurrent Protocol groups (i.e., -55%, -56%. and -55%, respectively).

## DISCUSSION

Identifying the best strategy for combining immune checkpoint inhibitors with other treatments to overcome resistance remains an ongoing challenge. We initially considered monitoring ctDNA as a surrogate for tumor dynamics, but unfortunately, none of the available kits we used have the sensitivity to generate meaningful data from mice and thus we stopped using ctDNA (data not shown). However, such a ctDNA assay may be useful in the future to better monitor the impact of treatments on tumor dynamics, provided that appropriate kits become available to detect murine ctDNA. The key role of CD8 T cells has been identified in predicting ICI efficacy and in characterizing immune-related adverse events (irAE)^[16]^. In addition, the presence of TILs has been shown to be associated with better clinical outcomes^[17]^. TILs contain different types of lymphocytes, including Tregs and CD8 T cells, which have opposing effects on tumor growth^[18]^. Therefore, monitoring of central (spleen), peripheral (blood) and tumor T cells is essential to better understand when to prioritize ICI.

Here, we observed that a larger tumor seemed to induce more CD45 cells and a stronger correlation was found with CD8 T cells. This correlation was maintained over time. This is consistent with the fact that tumor size plays a role in the tumor immune response. Baseline tumor size has been identified as a predictive biomarker for response to immunotherapy. Indeed, large tumor size may induce a higher tumor mutation burden (TMB) and therefore a better response^[19]^, although baseline tumor size is associated with poor prognosis. Notably, a study by Uehara *et al.* in 2022 showed that initial tumor size was not a relevant predictive factor in patients receiving a combination of ICI and chemotherapy^[20]^.

Early monitoring of B cells suggested an effect of tumor vasculature normalization induced by anti-VEGF on intratumoral B cell infiltration, as well as an effect of Pemetrexed during the first week of treatment. Focusing on T cells, the early increase observed with B cells was not found, except in the tumor for FD Cisplatin showing higher CD8 T cells and in the spleen for the control group showing higher CD3 T cells. The combination of anti-VEGF + CT groups induced higher CD8 and CD4 T cells in the spleen on Day 7. Higher CD8 T cells, but not CD4 T cells, were observed in the spleen of the anti-VEGF + CT group up to Day 14. However, we observed higher T CD4 cell levels in the blood for the anti-VEGF + CT, FD Pemetrexed and anti-VEGF groups. We first observed a central increase in CD4 T cells on Day 7, followed by a peripheral increase on Day 14.

This highlights some spatial immunomodulatory differences, suggesting significant spleen support in the anti-tumor immune response. For the T CD8/Treg ratio, early differences were seen only in the blood, with a lower ratio in both the FD Pemetrexed and anti-VEGF flat dose groups. On Day 14, the combination of anti-VEGF and CT showed a higher ratio, and overall, a two-week delay before adding the next ICI to this combination was considered optimal. As full-dose CT did not induce greater lymphopenia compared to the lower dose, this regimen (i.e., anti-VEGF followed by FD CT) was considered a test protocol to be further evaluated in the second trial. The test protocol group had significantly smaller tumors than all other groups on Days 21 and 26. In particular, the mean tumor size was significantly smaller than in the concurrent protocol, suggesting that the sequencing of the drugs may indeed play an important role in efficacy. When assessing tumor size as a percentage reduction relative to the control group, all treatments showed significant efficacy. However, the test protocol showed the best efficacy with a reduction of almost -71% on Day 26. Importantly, the fact that the reverse protocol performed slightly worse than the test protocol suggests that in addition to the order of the drugs, the order of the sequence is important to optimize the combination of CT and ICI.

As previously described by our group, anti-VEGF induces a transient phase of vascular normalization that increases drug delivery and improves efficacy^[9]^, highlighting the importance of drug scheduling when combining treatments. Here, we observed that similarly, the combination of anti-VEGF with Pemetrexed/Cisplatin doublet plus anti-PD-1 showed better efficacy when optimal sequencing was used, as tumor volume reduction improved from -55% with concurrent dosing to -71% with sequential dosing. Our current findings with cytotoxics echo previous experimental reports showing that sequencing targeted therapies with immune checkpoint inhibitors could also achieve higher efficacy^[21,22]^. In a previous study, we had already shown in BalbC mice grafted with KLN205 cells that the combination of anti-VEGF and the pemetrexed/cisplatin doublet led to an optimal remodeling of tumor immunity in a time-dependent manner, i.e., by best increasing the T CD8/Tregs ratio 2 weeks after administration, suggesting that sequential treatments with immunotherapy might be of interest, although at that time no efficacy study was performed to test this hypothesis^[23]^. Here, we shifted to an immune-refractory mouse model, i.e., C57BL/6 mice bearing LL/2 lung cancer cells. Indeed, LL/2 cells are derived from a Lewis lung carcinoma model that developed spontaneously in C57BL mice. In addition, these cells develop so-called “cold” lung cancer tumors with low infiltration of TILs and high infiltration of MDSC. This type of “cold” tumor may be the cause of ICI non-response and resistance observed in clinical practice. LL/2 cells have been utilized as a model to study resistance, particularly in optimizing the scheduling of Gemcitabine plus Cisplatin treatment^[24,25]^ and folate-based thymidylate synthase inhibitors such as Pemetrexed^[26]^. Our study suggests that ICIs overrule Cisplatin and Pemetrexed resistance of LL/2 cells and that scheduling anti-VEGF and cytotoxic therapy is essential to overrule resistance to ICIs due to “cold” tumors developed by LL/2 cells.

In this work, the two-week delay was confirmed and the hypothesis that sequential treatment might perform better than concurrent dosing was established. Indeed, in clinical practice, corticosteroids are used as antiemetics in the prevention of acute and delayed nausea and vomiting, induced by moderate to highly emetogenic chemotherapy (such as Cisplatin)^[27]^. Steroids are known to influence the efficacy of ICIs, mostly by inducing lymphopenia, even if this effect remains complex and unclear^[28]^. Our protocol shifts steroids (administrated on day 2 with Cisplatin) and anti-PD-1 treatment by 12 days, reducing conflicts between the lympholytic and immunosuppressive properties of steroids and ICIs.

Our experimental data show that our model was resistant to anti-PD1 as a single agent, that adding cytotoxics with anti-VEGF improved the efficacy, but that further sequencing treatments achieved better results eventually. However, there are several limitations to the current work: although the differences were found to be significant, they are from a single animal model of resistant NSCLC and would need to be further replicated in other models to be confirmed. In addition, as it will be difficult to decipher how drugs reshape tumor immunity in actual patients, mathematical translation of our findings into the clinical setting^[29]^ could help implement model-based dosing and scheduling of immunotherapy at the bedside. For example, several physiologically based pharmacokinetic models have been developed to describe the interaction of therapeutic agents with immune cells^[30]^ - potentially allowing inter-species extrapolation and better translation to the human setting. Replication of our findings in other preclinical settings should help to calibrate such a mathematical model with a view to in silico testing prior to conducting a comparative phase 2 study evaluating the performance of standard dosing versus model-guided dosing.

### Conclusion

The current trend in oncology is based on the development of precision medicine, i.e., moving away from trial-and-error practice to the development of model-based treatments. Although preliminary and only conducted in a single lung cancer animal model, our data suggest that standard concurrent dosing of cytotoxics and anti-PD-1 may not be an optimal strategy and that sequencing treatments at the bedside may achieve greater efficacy. To date, most attempts to combine immune checkpoint inhibitors with standard chemotherapy have been based on empirical designs - sometimes with successful results, sometimes with disappointing outcomes in terms of efficacy. Although our work is only experimental, it suggests that the same drugs, given the same dose, but in a different fashion, may be more effective.

## DECLARATIONS

### Copyright

© The Author(s) 2024.
