# Supplementary material for: Overcoming immuno-resistance by rescheduling anti-VEGF/cytotoxics/anti-PD-1 combination in lung cancer model
Source: Cancer Drug Resist. 2024 Mar 14;7:10. doi: 10.20517/cdr.2023.146 (PMC10951825; doi:10.20517/cdr.2023.146)
Supplement: Supplementary file 1 [file cdr-7-10-SupplementaryMaterials.pdf]

Sicard *et al. Cancer Drug Resist* 2024;7:10
DOI: 10.20517/cdr.2023.146

Cancer Drug Resistance

Supplementary Materials

Overcoming immuno-resistance by rescheduling anti-VEGF/cytotoxics/anti-PD-1 combination in lung cancer model

Guillaume Sicard[1], Dorian Protzenko[1], Sarah Giacometti[1], Fabrice Barlési[2,3], Joseph Ciccolini[1,4], Raphaelle Fanciullino[1,4]

[1]SMARTc Unit, CRCM Inserm U1068, Aix Marseille University, Marseille 13385, France.
[2]School of Medicine, Aix Marseille University, Marseille 13385, France.
[3]Department of Thoracic Oncology, Gustave Roussy Institute, Villejuif 94200, France.
[4]COMPO, CRCM Inserm U1068 INRIA, Marseille 13385, France.

Correspondence to: Prof. Joseph Ciccolini, COMPO, CRCM Inserm U1068 INRIA, 27 Bd Jean Moulin, Marseille 13385, France. E-mail: joseph.ciccolini@univ-amu.fr

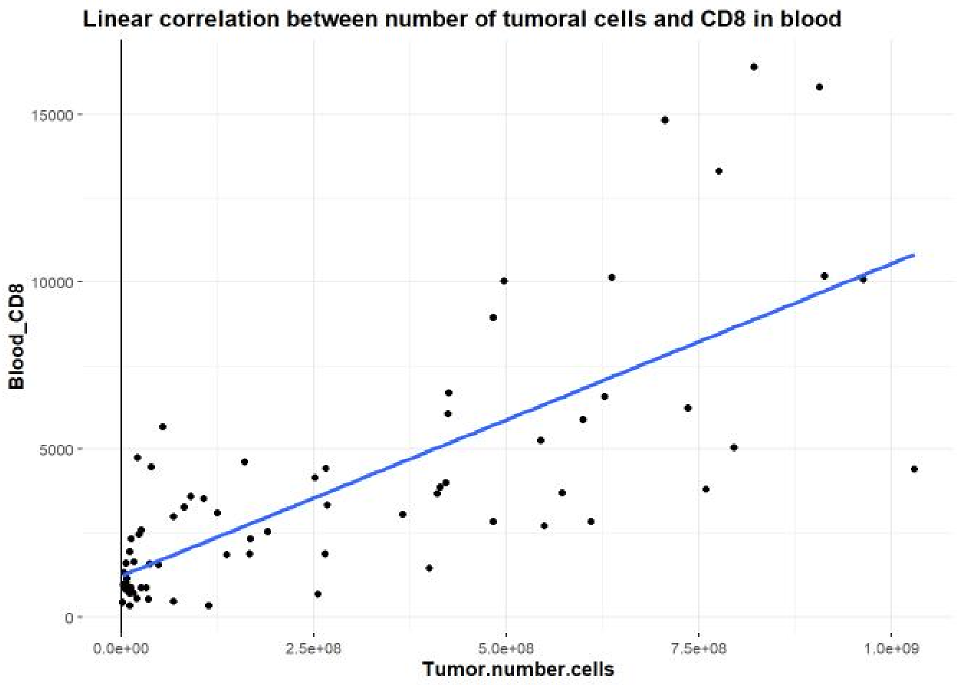

Supplementary Figure 1. Correlation between tumor cells count and CD8 T cells in blood. In Supplementary Figure 1, a strong linear correlation (r = 0.75) was found between CD8 T cells in blood and tumor number cells ($P < 0.001$).

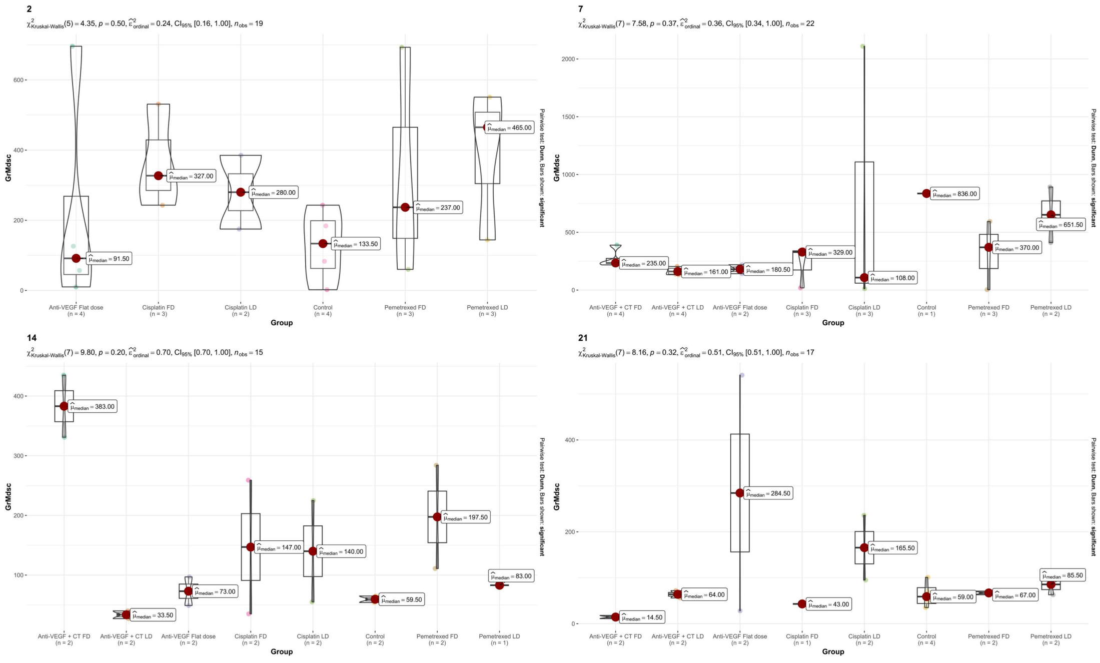

Supplementary Figure 2A. GrMDSC monitoring in tumor.

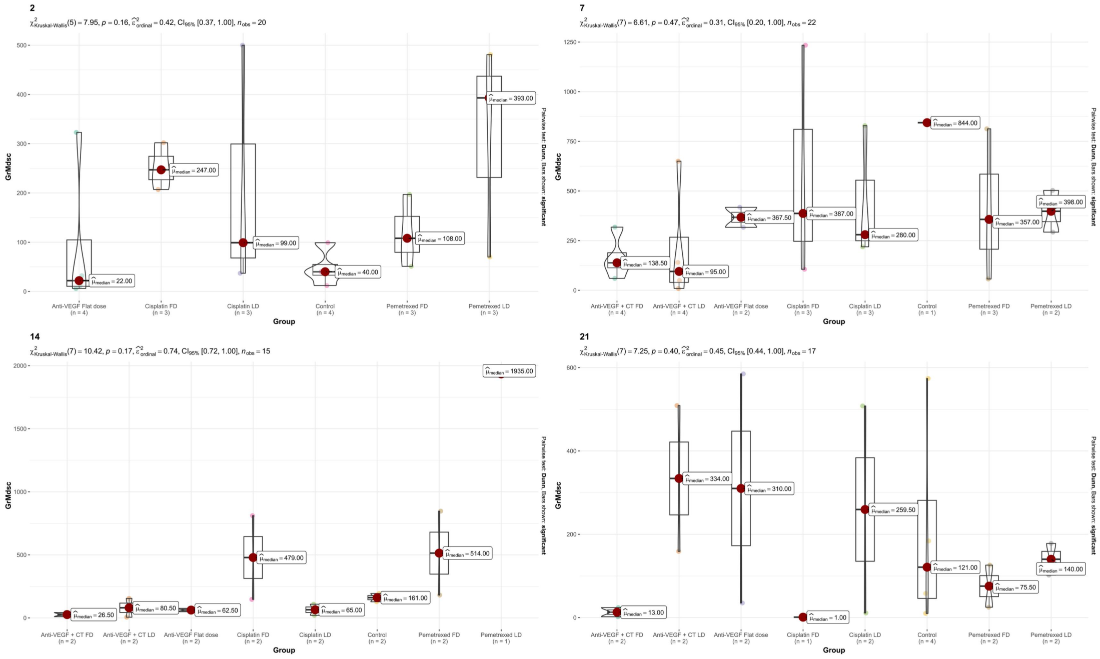

Supplementary Figure 2B. GrMDSC monitoring in spleen.

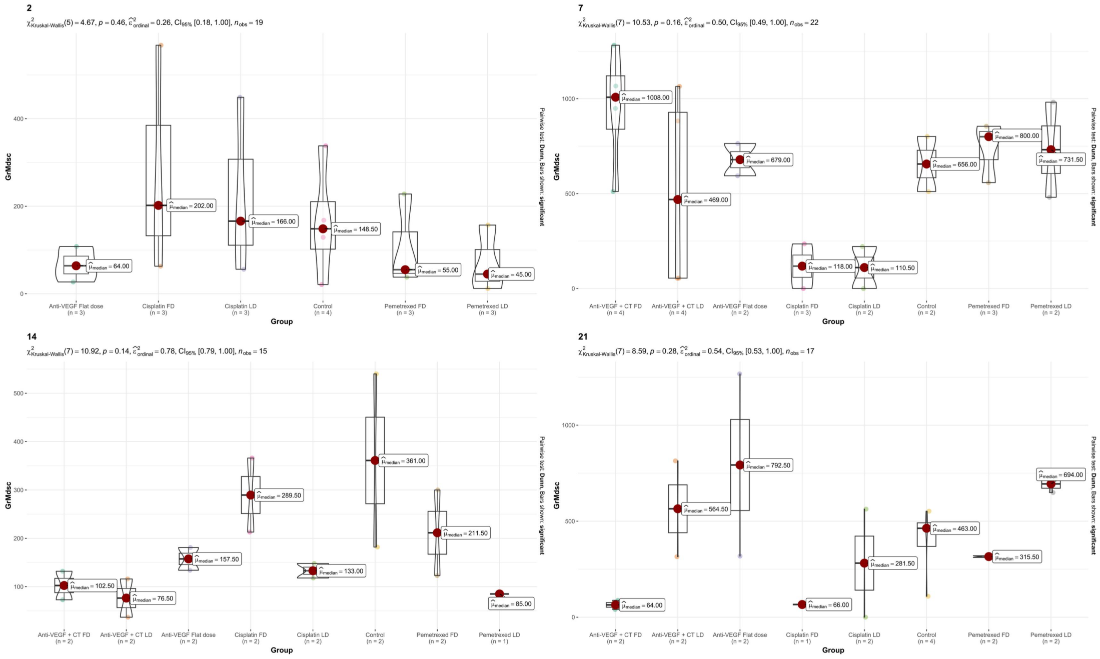

Supplementary Figure 2C. GrMDSC monitoring in blood.

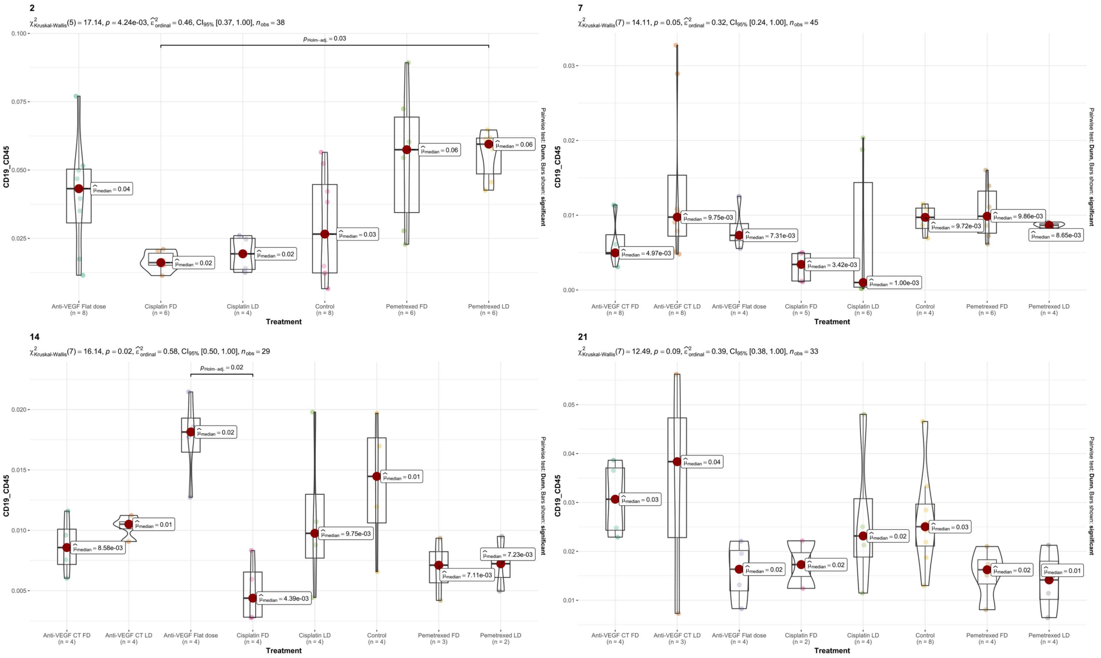

Supplementary Figure 3A. B cells (CD19+) monitoring in tumor.

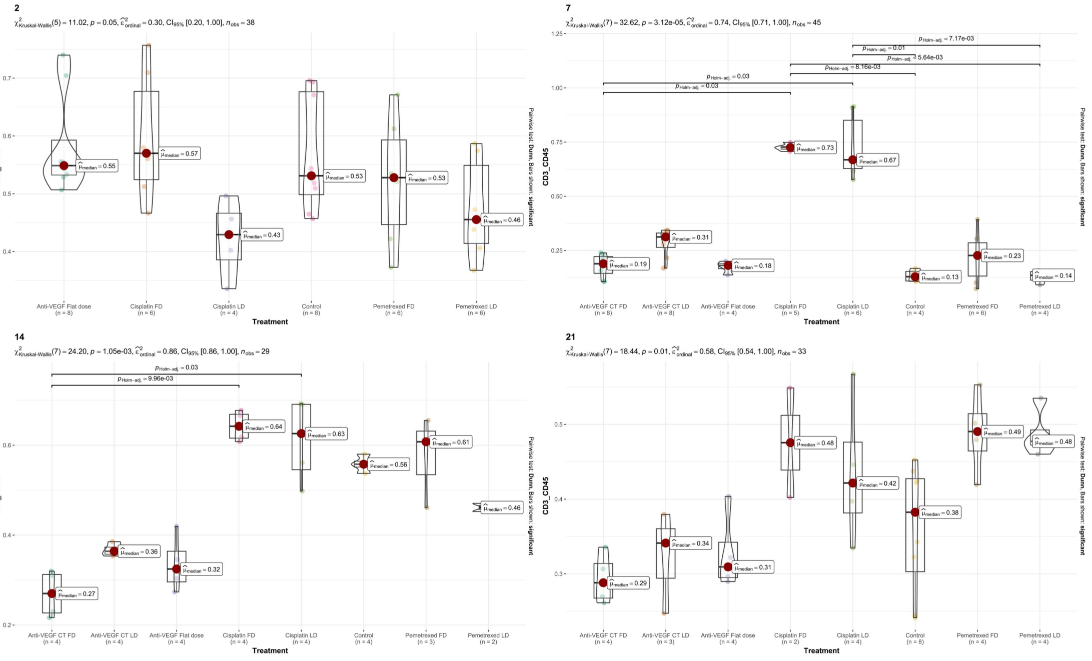

Supplementary Figure 3B. CD3 T cells monitoring in tumor.

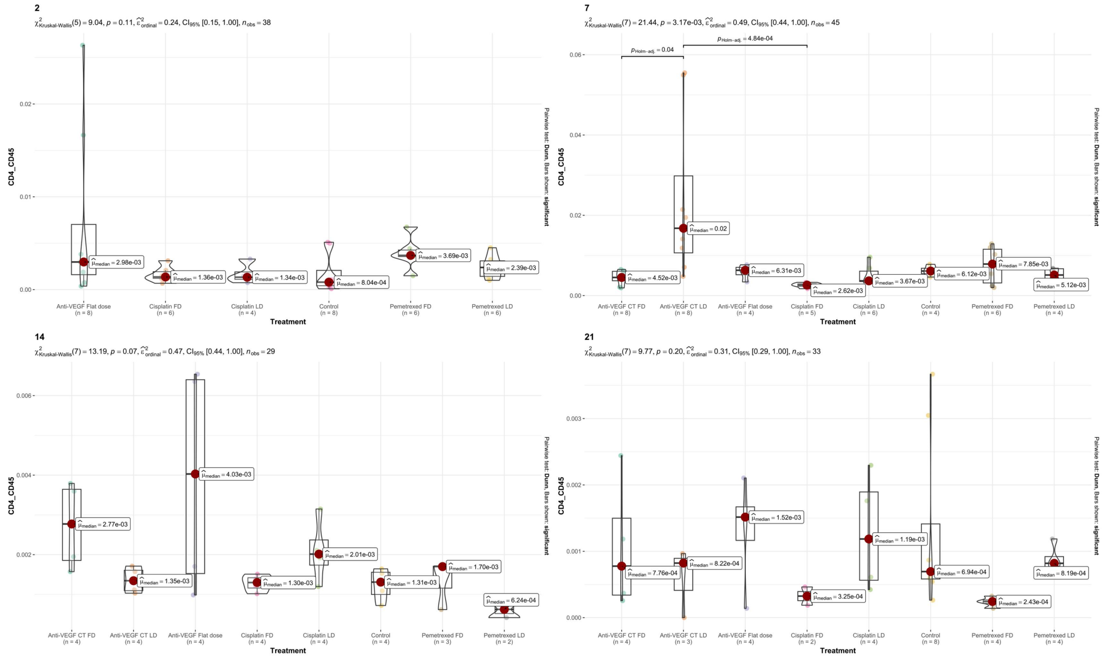

Supplementary Figure 3C. CD4 T cells monitoring in tumor.

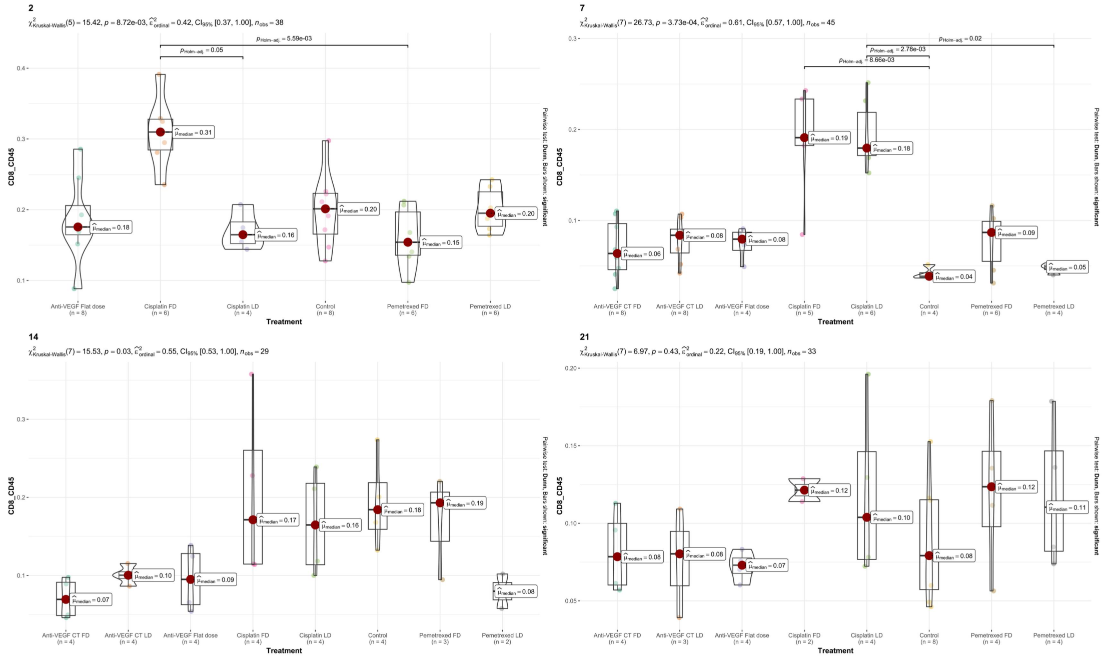

Supplementary Figure 3D. CD8 T cells monitoring in tumor.

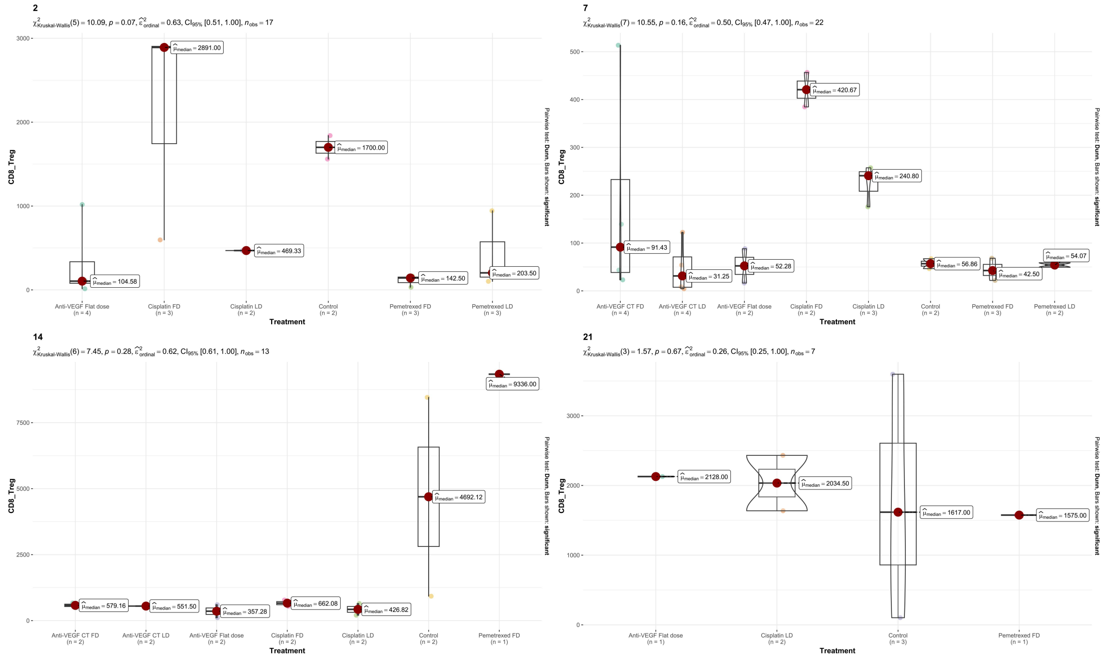

Supplementary Figure 4. T CD8/Treg ratio monitoring in tumor.

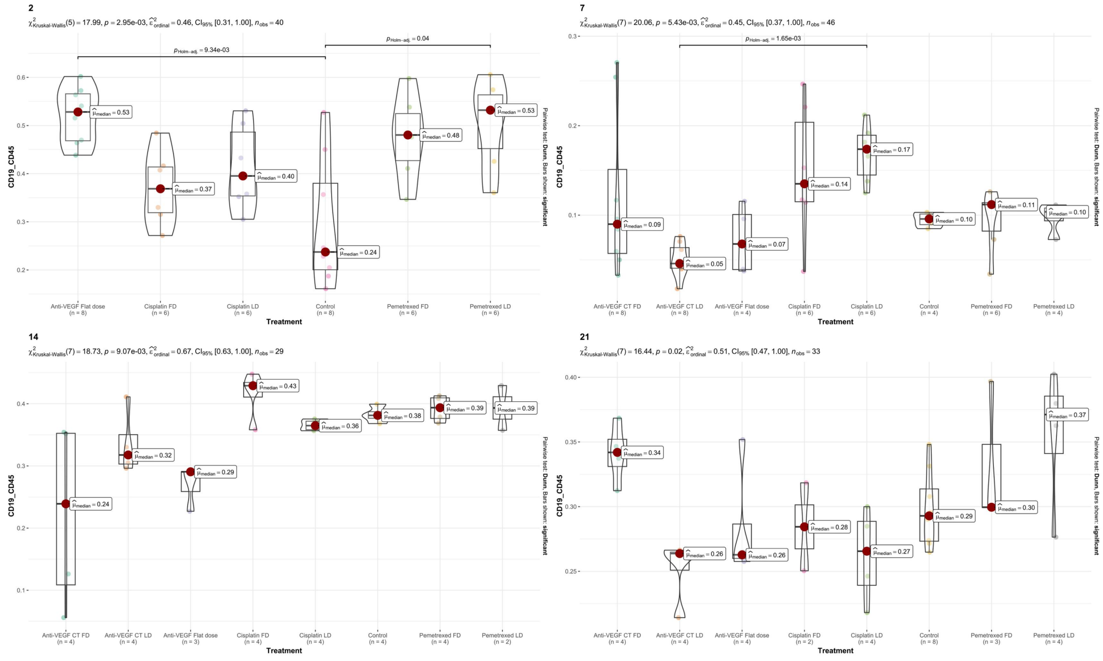

Supplementary Figure 5A. B cells (CD19+) monitoring in spleen.

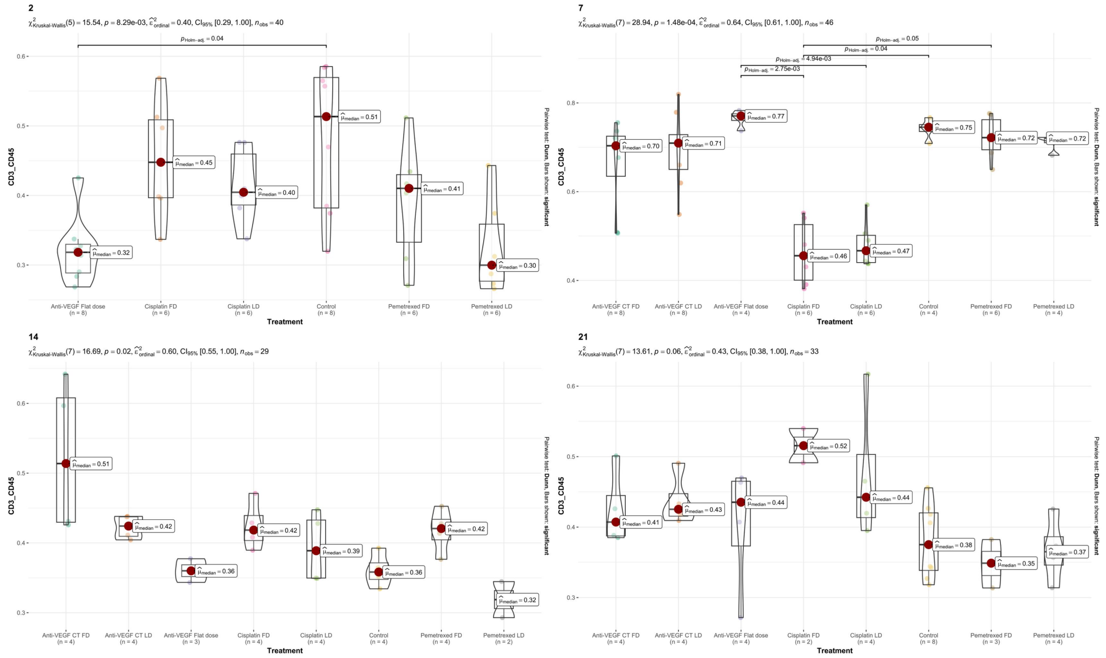

Supplementary Figure 5B. CD3 T cells monitoring in spleen.

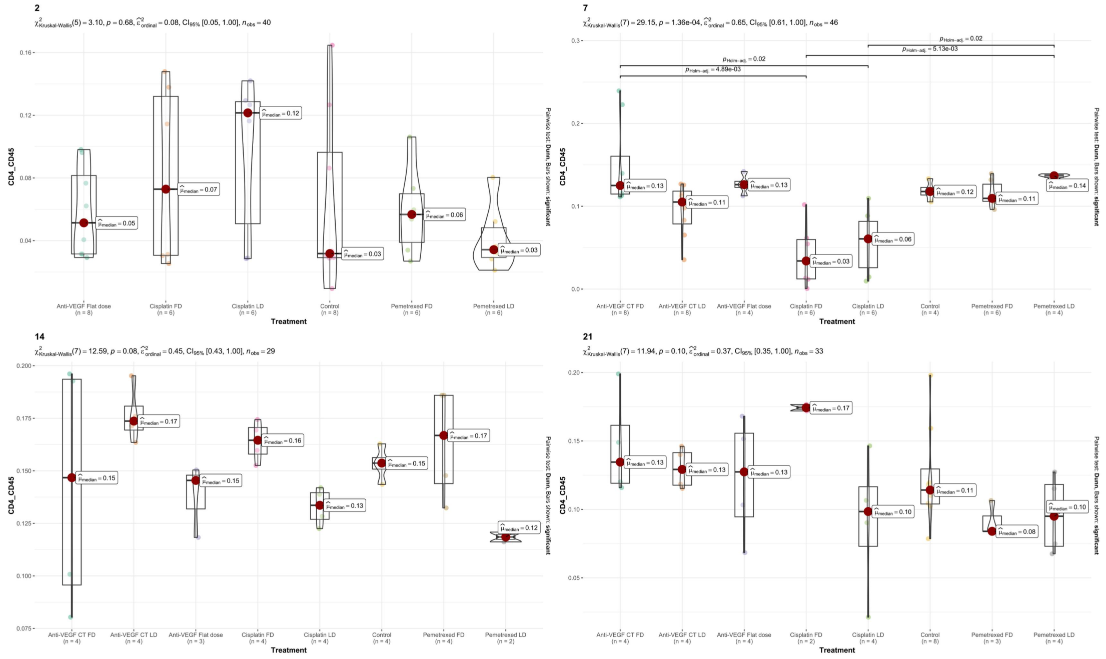

Supplementary Figure 5C. CD4 T cells monitoring in spleen.

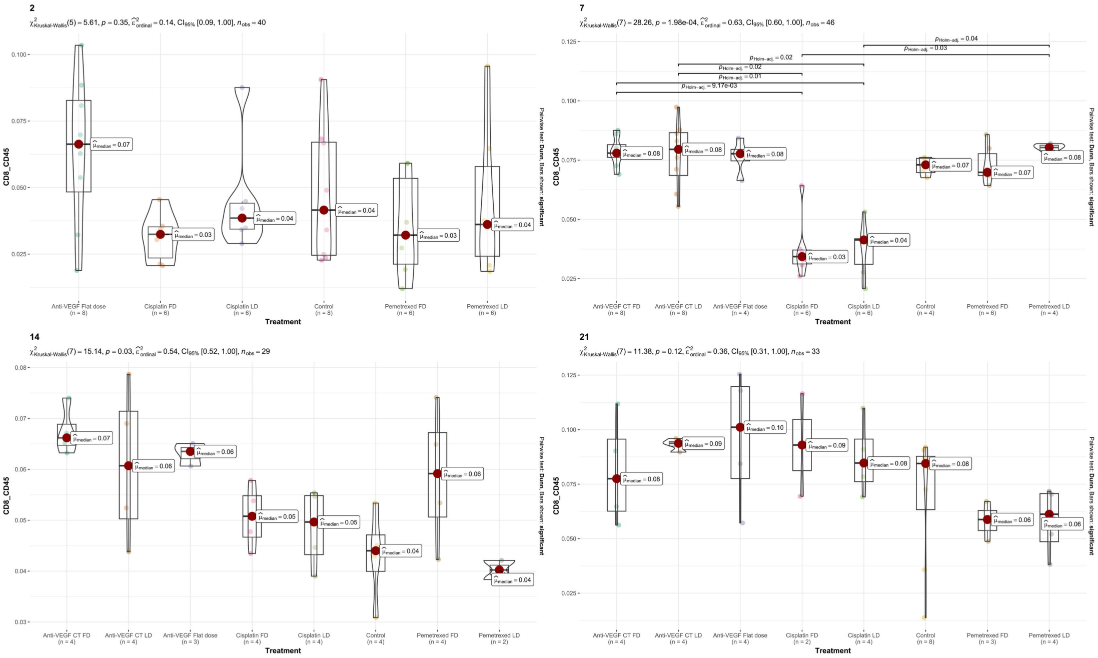

Supplementary Figure 5D. CD8 T cells monitoring in spleen.

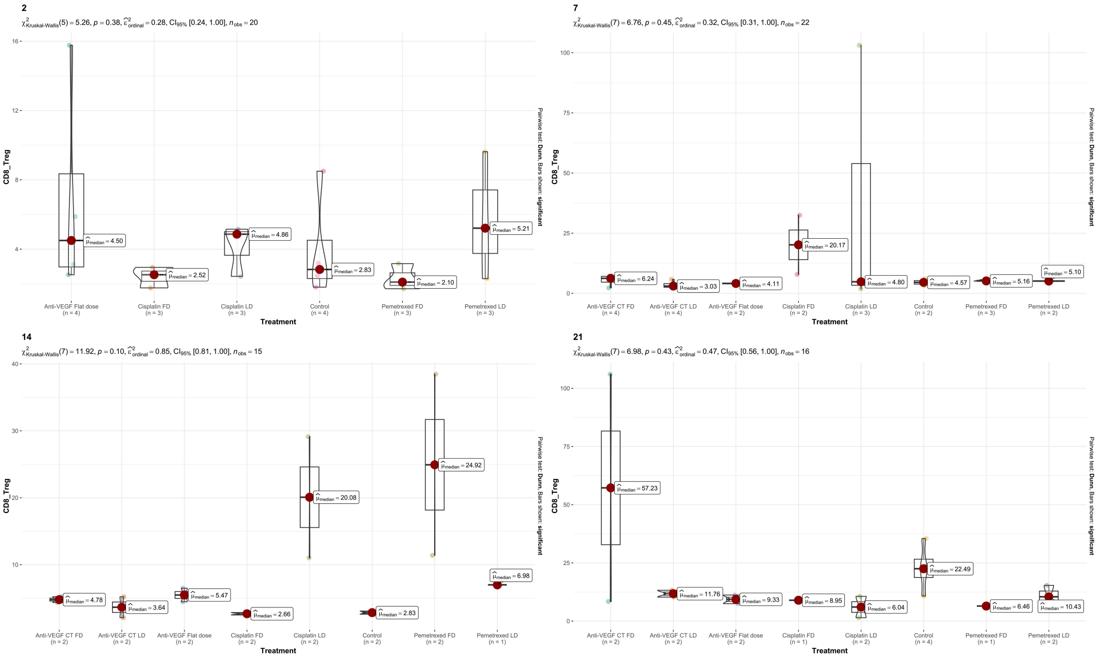

Supplementary Figure 6. T CD8/Treg ratio monitoring in spleen.

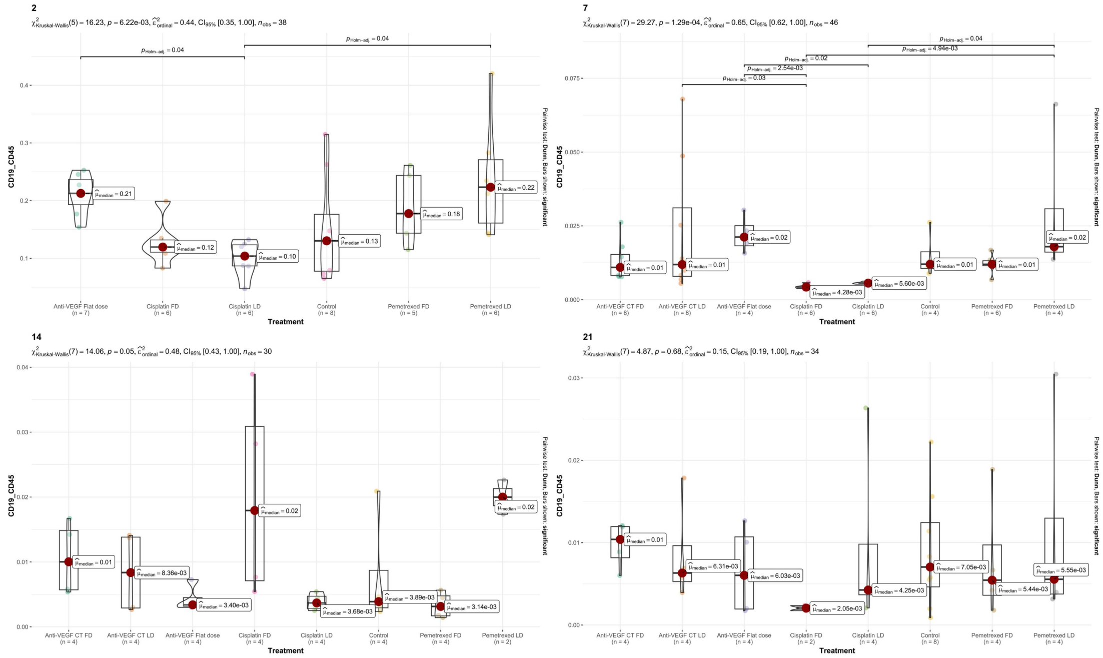

Supplementary Figure 7A. B cells (CD19+) monitoring in blood.

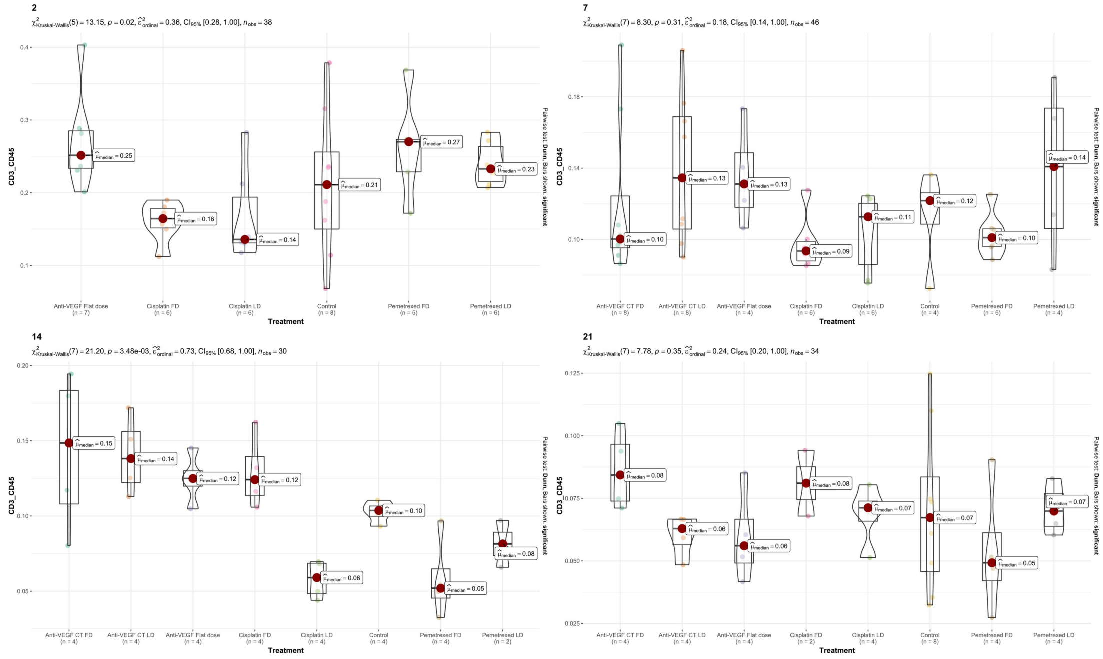

Supplementary Figure 7B. CD3 T cells monitoring in blood.

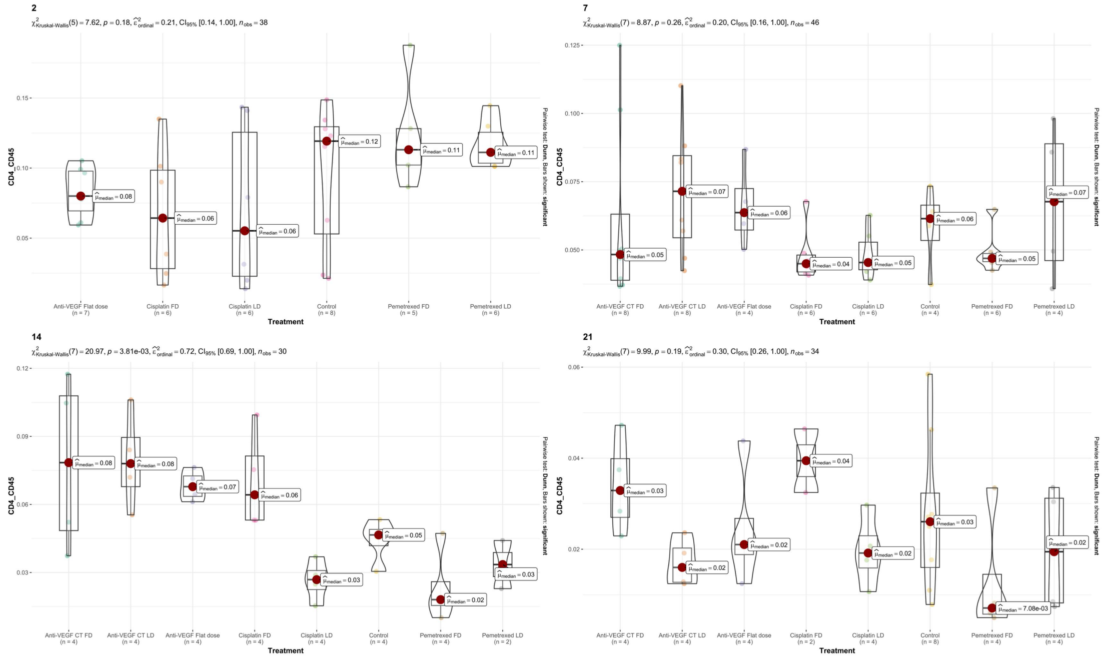

Supplementary Figure 7C. CD4 T cells monitoring in blood.

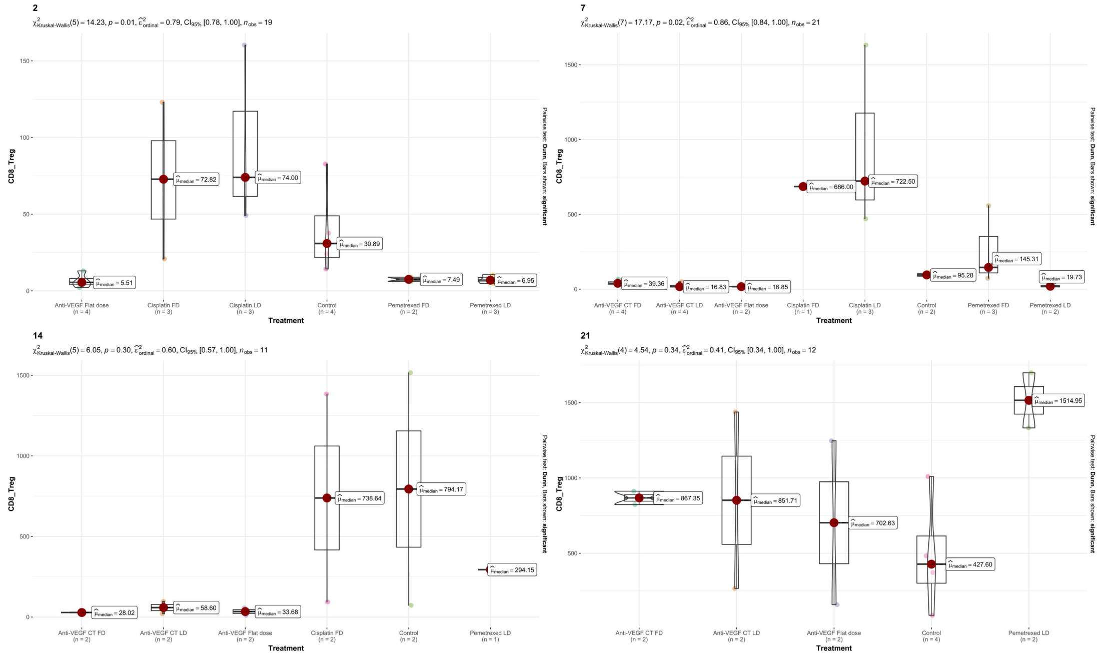

Supplementary Figure 8. T CD8/Treg ratio monitoring in blood.
